# REVIEW: Towards a systems approach for understanding honeybee decline: a stocktaking and synthesis of existing models

**DOI:** 10.1111/1365-2664.12112

**Published:** 2013-06-10

**Authors:** Matthias A. Becher, Juliet L. Osborne, Pernille Thorbek, Peter J. Kennedy, Volker Grimm

**Affiliations:** ^1^ Rothamsted Research West Common Harpenden AL5 2JQ UK; ^2^ Syngenta Environmental Safety Jealott's Hill International Research Centre Bracknell RG42 6EY UK; ^3^ UFZ Helmholtz Centre for Environmental Research – UFZ Permoserstr. 15 04318 Leipzig Germany; ^4^ Institute for Biochemistry and Biology University of Potsdam Maulbeerallee 2 14469 Potsdam Germany; ^5^ Environment & Sustainability Institute University of Exeter Cornwall Campus Penryn TR10 9EZ UK

**Keywords:** *Apis mellifera*, colony decline, feedbacks, integrated model, multiple stressors, predictive systems ecology, review

## Abstract

1. The health of managed and wild honeybee colonies appears to have declined substantially in Europe and the United States over the last decade. Sustainability of honeybee colonies is important not only for honey production, but also for pollination of crops and wild plants alongside other insect pollinators. A combination of causal factors, including parasites, pathogens, land use changes and pesticide usage, are cited as responsible for the increased colony mortality.
2. However, despite detailed knowledge of the behaviour of honeybees and their colonies, there are no suitable tools to explore the resilience mechanisms of this complex system under stress. Empirically testing all combinations of stressors in a systematic fashion is not feasible. We therefore suggest a cross‐level systems approach, based on mechanistic modelling, to investigate the impacts of (and interactions between) colony and land management.
3. We review existing honeybee models that are relevant to examining the effects of different stressors on colony growth and survival. Most of these models describe honeybee colony dynamics, foraging behaviour or honeybee – varroa mite – virus interactions.
4. We found that many, but not all, processes within honeybee colonies, epidemiology and foraging are well understood and described in the models, but there is no model that couples in‐hive dynamics and pathology with foraging dynamics in realistic landscapes.
5. *Synthesis and applications*. We describe how a new integrated model could be built to simulate multifactorial impacts on the honeybee colony system, using building blocks from the reviewed models. The development of such a tool would not only highlight empirical research priorities but also provide an important forecasting tool for policy makers and beekeepers, and we list examples of relevant applications to bee disease and landscape management decisions.

## Abstract 2

We describe how a new integrated model could be built to simulate multifactorial impacts on the honeybee colony system, using building blocks from the reviewed models. The development of such a tool would not only highlight empirical research priorities but also provide an important forecasting tool for policy makers and beekeepers, and we list examples of relevant applications to bee disease and landscape management decisions.

## Introduction

Whilst global stocks of managed honeybee colonies appear to be increasing (Aizen & Harder 2009), substantial regional losses have been documented (Stokstad 2007; Pettis & Delaplane 2010; Potts *et al*. 2010). There is serious concern about whether stocks are sustainable (Ellis, Evans & Pettis 2010) and able to service rising demand for insect‐pollinated produce caused by changing human diets (Aizen *et al*. 2008) and also whether this will affect wild flower pollination and hence biodiversity. Despite a plethora of publications and debate about the status of honeybee health across the world, knowledge of what is driving colony losses is still elusive (Ratnieks & Careck 2010), and there is growing demand for tools that can be used to predict the consequences of different hive and landscape management practices on colony survival (Ratnieks & Careck 2010; EFSA 2012; Osborne 2012).

Extensive research has been dedicated to identifying single factors that might drive the decline, for example, varroa (*Varroa destructor* Anderson and Trueman) mites (Le Conte, Ellis & Ritter 2010; Rosenkranz, Aumeier & Ziegelmann 2010), pathogens (i.e. bee viruses and *Nosema spp*.; Higes *et al*. 2009; Cox‐Foster *et al*. 2007; Genersch & Aubert 2010), pesticides residues (Thompson 2003; Alaux *et al*. 2010; Johnson *et al*. 2010; Henry *et al*. 2012) and beekeeping practices (Oldroyd 2007). However, there is a growing consensus that the decline in honeybee health is caused by a combination of factors (e.g. vanEngelsdorp *et al*. 2009; Ratnieks & Careck 2010). Instead of a single factor, multiple stressors might be responsible for increased colony mortality and a solution to ensuring sustainable populations is likely to require a concerted effort targeting several of the causes, which might have interactive effects.

However, gaining understanding through multifactorial empirical approaches is likely to be immensely time‐ and resource‐consuming and the results challenging to interpret. Other approaches that can supplement experimental approaches are urgently needed (EFSA Panel on Plant Protection Products & their Residues (PPR) 2012; Osborne 2012). Cross‐level ‘systems ecology’, based on mechanistic modelling (Evans, Norris & Benton 2012; Grimm & Railsback 2012), is such an approach. It incorporates not only processes at individual and colony level, but also the ‘top down’ and ‘bottom up’ regulating mechanisms and the interactions between associated organisms (e.g. parasites and pathogens) that affect bee behaviour and health. Following this approach would lead to an integrated model that takes into account multiple processes and factors, acting at different levels and scales, to predict overall colony strength, survival and behaviour in heterogeneous landscapes.

There already exists a wide range of honeybee models (for an overview, see Schmickl & Crailsheim 2007). Here, we present a review of models that target different components of the system, with the following three aims: first, by reviewing and assessing existing models, we obtain a comprehensive overview of the conceptual understanding of honeybee colony dynamics that is represented in these models. Secondly, since existing models reflect the current understanding of various aspects of honeybees, we scan them for designs, submodels and parameter values that can be used as building blocks for integrated models that explore honeybee dynamics and mortality. Thirdly, we outline what such an integrated model could look like and assess to what degree this is a suitable approach to explore honeybee decline.

In our review, we distinguished between three main categories of models, which address within‐hive colony dynamics (referred to as ‘colony models’ in the following), interaction between honeybees and varroa mites (‘varroa models’) and foraging in a heterogeneous and possibly dynamic landscape (‘foraging models’). We choose these categories because within‐hive dynamics and foraging are essential elements of honeybee dynamics, and varroa mites are generally believed to be an essential stressor. Nevertheless, we also scanned the literature for models that address further stressors, including pesticides and pathogens. We restricted our search to models of single colonies and omitted population or metapopulation models.

More generally, we consider our synthesis of honeybee models as an example of how such a cross‐level systems approach could be initiated for any ecological system to understand resilience mechanisms and response to multiple stressors. Finally, stakeholders (such as beekeepers, policy makers and landowners) are spending considerable resources on developing management strategies, both targeted at the hive and the landscape, to improve honeybee health and survival. We discuss how such an integrated model would provide a tool to explore the impact of these different options such that the most effective strategies can be implemented.

## Materials and methods

We searched the literature for dynamic models that address honeybees and analysed all models that are potentially relevant for understanding honeybee colony survival and death. Articles were chosen using the ‘Web of Science’ section of the data base ISI Web of Knowledge (http://pcs.isiknowledge.com). We searched separately for colony and foraging models. We started with year 1989 because in this year, DeGrandi‐Hoffman *et al*. (1989) published the pioneering BEEPOP model. Criteria used for colony models were as follows: Topic = ((honeybee OR honey bee OR Apis) AND (population dynamics OR colony growth) AND (model* OR simulation) AND (colony OR hive OR varroa OR simulation model OR Nosem* OR pesticide* OR beekeep* OR genetic* OR bacteria* OR virus OR viral OR pathogen)). We included ‘colony OR varroa’ because often varroa models were linked with colony models. Additionally, we took into account our own collection of honeybee model publications, both to check the detection power of our data base scan and to include models from publications that were not detected by our combination of keywords. We searched for models of single colonies of honeybees, which include the bee's full life cycle and represent colony dynamics long enough, in principle, to predict colony persistence or extinction. Likewise, we searched for varroa models that include the mite's full life cycle and run long enough to explore their effect on honeybee colony development.

The search for foraging models was conducted in a similar way but based on the following search criteria: Topic = ((honeybee OR honey bee OR Apis) AND (scout OR forag* OR recruitment OR danc*) AND (nectar OR pollen OR food) AND (movement OR flight OR search OR pattern) AND (landscape OR patches OR structure OR flowers OR fields) AND (model* OR simulation)). We excluded models focusing on pollination or only on specific aspects of foraging.

Of the chosen models, we checked for their purpose, entities and their state variables and processes (Grimm *et al*. 2006, 2010). For the latter, we particularly checked for feedbacks built into the models, that is, processes depending on the current or past state of the model's entities. Additionally, we checked what model outputs were presented and to what degree models were analysed; the latter included three aspects: simulation experiments to better understand how model results emerge, sensitivity analyses of the model output to changes in parameters and robustness analyses, here defined as exploring sensitivity in model output to changes in model structure.

## Results

We identified eight colony models, 11 varroa models and 12 foraging models as being relevant for our review (Table 1). 13 relevant publications were found in our collections that were not detected by the data base search (referred to as ‘additional’ in Table S1). We did not find references to further relevant models in the publications we reviewed. In Table S1 (Supporting Information), we list and briefly describe further honeybee models found in other publications that were not included in our analyses, and we give reasons for their exclusion.

**Table 1:** Honeybee models evaluated in this review. For forager models, ‘se’, test of nectar source selection as in Seeley, Camazine & Sneyd 1991. Details of model output and structure are included in Table S2, Supporting Information

Model type	Reference	Purpose of model	R	S	V
C	Omholt 1986	Explain brood‐rearing peaks in nonswarming colonies	–	+	+
C	DeGrandi‐Hoffman *et al*. 1989	Simulate honeybee population dynamics to support beekeeping management	–	+	+
C	Martin 2001, a	Explain the link between varroa mite infestation and honeybee colony death, including the effects of virus diseases	–	+	+
C	Al Ghamdi & Hoopingarner 2004, a	Develop a tool for bee research; explore interaction between varroa and honeybees	–	–	–
C	Thompson *et al*. 2005, 2007, b	Explore effect of an insecticide on colony dynamics	–	–	–
C	Schmickl & Crailsheim 2007	To create a colony model that includes important feedback loops, pollen supply and brood cannibalism	–	++	++
C	Becher *et al*. 2010	Influence of temperature during development on colony survival	–	+	–
C	Khoury, Myerscough & Barron 2011	Impact of increased forager mortality on colony growth and development	+	–	–
V	Omholt & Crailsheim 1991	Tool for estimating varroa infestation in winter by death rates in autumn to decide whether a treatment is necessary	–	–	+
V	Calis, Fries & Ryrie 1999b (an update and extension of Fries, Camazine & Sneyd 1994)	Explore interaction of honeybee and mite population and the effects of mite resistance, beekeeping techniques and control treatments	–	+	–
V	Fries, Camazine & Sneyd 1994 (predecessor of Calis, Fries & Ryrie 1999b)	Population dynamics of varroa, impact of varroa treatment	+	+	–
V	Boot *et al*. 1995	Study the circumstances under which specialization on drone brood would be a better strategy than reproduction in both types of cell	–	+	+
V	Martin 1998 (predecessor of Martin 2001, c)	To understand why varroa mites have become a serious problem, to advice beekeepers and to provide a tool for researchers	–	–	+
V	Calis, Boot & Beetsma 1999a	Test effectiveness of different methods to trap mites in brood combs	–	–	–
V	Wilkinson & Smith 2002	To study varroa population dynamics, monitoring methods and biological control methods	–	++	–
V	DeGrandi‐Hoffman & Curry 2004 (extension of BEEPOP)	Predict the influence of varroa on honeybee colony population growth and survival under different weather conditions, miticides and immigration of mites	–	–	+
V	Sumpter & Martin 2004	To determine the mite load that will cause a virus epidemic resulting in a colony collapse; influence of hygienic behaviour and division of labour	–	+	–
V	Vetharaniam & Barlow 2006 (based on Wilkinson & Smith 2002)	To explore the use of a benign varroa haplotype as biocontrol for a virulent haplotype	–	+	–
V	Vetharaniam 2012	To predict varroa reproduction rate, based on a single equation	–	–	–
F	Schmid‐Hempel, Kacelnik & Houston 1985	Comparison of energy delivery rate with energetic efficiency as currencies to explain partially filled crops of foragers	–	+	+
F	Camazine & Sneyd 1991	Demonstrate how collective foraging patterns emerge from the behaviour of individual bees	–	+	+ se
F	De Vries & Biesmeijer 1998; 2002	Obtain a set of rules that is necessary and sufficient for the generation of the collective foraging behaviour	–	+	+
F	Dukas & Edelstein‐Keshet 1998	Predict spatial distribution of solitary and social foragers that share nesting aggregation using three currencies	–	–	–
F	Sumpter & Pratt 2003	Review of previous differential equation models of foraging and recruitment and formulation of general framework that incorporates them all, with case studies for ants and honeybees	–	–	+ se
F	Higginson & Gilbert 2004	Explore if energy profit per wingbeat is a currency that can explain foraging behaviour	–	+	+
F(HoFoSim)	Schmickl & Crailsheim 2004	Simulation of collective foraging on basis of decentralized foraging decision system	–	–	+ se
F	Dornhaus *et al*. 2006a	Quantify the benefits of recruitment under different spatial distributions of nondepleting resource patches and with different colony sizes	–	++	–
F	Dornhaus *et al*. 2006b	How much time should a forager spend in a patch, if a superior patch may become available?	–	–	–
F	Beekman *et al*. 2007	Explore mechanisms by which colony regulates N scouts in relation to N recruits	–	–	++
F	Johnson & Nieh 2010	Test whether the ‘stop signal’ provides a benefit when high costs are associated with waggle dance	–	–	+
F(HoFoReSim)	Schmickl, Thenius & Crailsheim 2012	Extension of HoFoSim by implementing receiver bees as agents	–	–	+ se

C, colony model; V, varroa model; F, foraging model; R, robustness analysis (exploring alternative formulations of submodels); S, sensitivity analysis (local sensitivity analysis of several parameters or sensitivity experiments where one parameter was varied over a larger range); V, verification (comparison of model output to observations); ‘–’, none; ‘+’, some limited effort; ‘++’, considerable effort.

Model very similar to DeGrandi‐Hoffman *et al*. (1989).

a Honeybee model very similar to DeGrandi‐Hoffman *et al*. (1989) and varroa model very similar to Fries, Camazine & Sneyd (1994).

b Model very similar to Wilkinson & Smith (2002).

c Martin 2001 includes a fully developed varroa model, but is filed under colony models (Tables 1 and 2). Colony dynamics emerges from a fully developed colony model.

### Colony models

**Figure 1:**
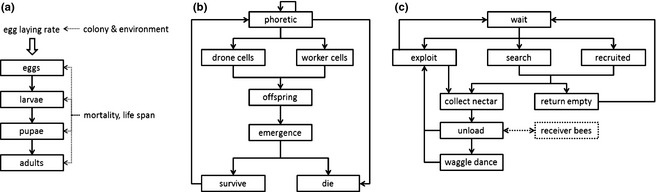
Schematic overview of main processes in honeybee models. (a) Colony models: based on an egg‐laying rate, bees pass through the developmental stages of eggs, larvae, pupa and adults, with a specific mortality acting on each of these stages. Some models distinguish between workers and drones, others only simulate workers. (b) Varroa models: phoretic mites (i.e. carried by bees) invade drone or worker cells, reproduce, emerge together with the adult bees, face the risk to die by falling from the comb and finally join again the group of phoretic mites. (c) Foraging models: the main processes of foraging models include waiting in the hive, searching for a nectar source, collect nectar if successful, unload nectar back in the colony (which might require receiver bees) and recruit new bees.

A summary of the main processes relevant for the structure and dynamics of a honeybee colony is provided in Fig. 1a. In principle, all colony models include these processes, but the simpler models aggregate some of them, whereas the more complex ones use submodels to describe the main processes in more detail. Accordingly, colony models range from very simple to very complex, both regarding structure (Table S3) and processes (Table S4). Three models are highly aggregated and compile the number of individuals of different life stages or categories into a single entity (Omholt 1986; Thompson *et al*. 2005, 2007; Khoury, Myerscough & Barron 2011). All other models additionally consider the age of individuals in some or all categories, that is, eggs, larvae, pupae, in‐hive bees and foragers. They are thus age‐based cohort models in which time and thus age proceed in steps of one day; changes in cohort size are described with difference equations.

The design of these age‐structured models is influenced by one of the first model of this kind, BEEPOP (DeGrandi‐Hoffman *et al*. 1989). Cohort dynamics are driven by egg laying of the queen, which in turn in most models is driven by weather parameters or an imposed seasonal unimodal function with a peak in summer (Table S4). HoPoMo (Schmickl & Crailsheim 2007) is, with 65 equations, the most complex colony model. HoPoMo ignores the age structure of adult bees and focuses instead on their daily dynamic allocation to tasks (nursing, food processing, foraging), which is driven by available stores of pollen, nectar and honey and by the demand of the colony for these resources, which in turn depends on the current size and structure of the colony (Tables S3 and S4).

Only four models include effects of stressors. Martin (2001) and Al Ghamdi & Hoopingarner (2004) link the bee colony to varroa mites and their effects. Thompson *et al*. (2005, 2007) address the impact of a certain pesticide (insect growth regulator) on colony size, and Henry *et al*. (2012) and Cresswell & Thompson (2012) use Khoury, Myerscough & Barron's (2011) model or a modified version of it, to predict behavioural effects of an insecticide on colony survival. No colony model represents foraging explicitly or considers a combination of varying numbers of foragers and mites or pesticides.

Model analysis was generally found to be quite limited, focusing on census dynamics or the sensitivity of peak population size to one or a few parameters (Table S2). More in‐depth analyses are presented by Martin (2001) and Schmickl & Crailsheim (2007), with the latter providing the most comprehensive evidence of structural realism (Grimm & Railsback 2012), that is, that the model is able to simultaneously reproduce an entire set of empirical patterns, observed at different levels of organization and different scales.

### Varroa models

Varroa population dynamics is driven by structure and dynamics of the infested colony (Fig. 1b). The varroa models therefore mainly differ in their representation of the honeybee colony, ranging from a mere probability of finding a drone or worker brood cell (Boot *et al*. 1995) to an independent, fully fledged honeybee model (DeGrandi‐Hoffman & Curry 2004; see also Martin 2001). The simpler varroa models including honeybee colony dynamics do not represent interactions between varroa and honeybees (Omholt & Crailsheim 1991; Fries, Camazine & Sneyd 1994; Boot *et al*. 1995; Martin 1998; Calis, Boot & Beetsma 1999a; Calis, Fries & Ryrie 1999b; Wilkinson & Smith 2002; Vetharaniam & Barlow 2006), that is, mite infestation does not affect brood survival and colony dynamics. The first model including a feedback between mite infestation and colony dynamics (Martin 2001) is based on an earlier varroa model (Martin 1998) linked to an adapted version of BEEPOP (DeGrandi‐Hoffman *et al*. 1989). Here, mites act as vector for a virus (APV: acute paralysis virus or DWV: deformed wing virus), which affects the life span of infected bees, represented via empirical survivorship curves. A similar approach with the same viruses is taken by Sumpter & Martin (2004); however, life span of healthy and infected bees is determined by a daily mortality rate and the mite population is constant. DeGrandi‐Hoffman & Curry (2004) extended their BEEPOP model to include varroa dynamics. They did not simulate virus dynamics but instead assumed that mite invasion into the brood cells itself reduces the life span of the affected bees, depending on the number of invaded mites.

With the exception of Omholt & Crailsheim (1991), all models distinguish between the phoretic phase of the mite's life cycle (i.e. being attached to adult bees) and the reproductive phase in the bee's brood cells. All models also distinguish between reproduction in worker and drone cells, as varroa mites not only invade drone cells preferentially but also produce more offspring in drone cells.

Several models address the possibilities of mite control including temporary application of organic acids or acaricides (Fries, Camazine & Sneyd 1994; Martin 1998, 2001; Calis, Fries & Ryrie 1999b; DeGrandi‐Hoffman & Curry 2004), culling of drone brood (Fries, Camazine & Sneyd 1994; Calis, Boot & Beetsma 1999a; Calis, Fries & Ryrie 1999b; Sumpter & Martin 2004) and temporary queen removal to create broodless conditions (Calis, Boot & Beetsma 1999a; Calis, Fries & Ryrie 1999b). Vetharaniam & Barlow (2006) explore the possibility of suppressing the virulent varroa haplotype by inoculation of a benign varroa haplotype.

### Foraging models

Foraging includes several processes, so variation in model structure is larger than for varroa models. Typically, the models contain the following entities and processes: A workforce of unemployed foragers is waiting in the hive (Fig. 1c). Some of the foragers may start to search for a food source as scouts (if they are not informed) or as recruits (if they were informed by following a waggle dance). If they find a food source, they fill their crop and return to the colony and become experienced foragers. After unloading, they may dance and recruit more foragers to this specific food source. Foragers can abandon their food source and search for a new one or abandon foraging and become unemployed.

The foraging models reviewed focus on the processes of food collection and worker bee allocation to one or several food sources. Food sources represented in the models are feeders with sugar solution or natural patches with nectar providing flowers. The workforce, that is, the number of available foragers, is assumed to be constant in all models and thus not linked to colony dynamics. Where time is represented via discrete time steps, these are short (0·5–36 s) and simulations usually cover a few hours.

Some models require receiver bees for successful foragers to unload their nectar (De Vries & Biesmeijer 2002; Schmickl & Crailsheim 2004; Johnson & Nieh 2010; Schmickl, Thenius & Crailsheim 2012). High foraging activities can then lead to queues of bees waiting for a nectar receiver. All these models also contain ‘tremble dances’, which can be performed by successful foragers to activate receiver bees.

Mortality during foraging is only taken into account by a few models (Dukas & Edelstein‐Keshet 1998; Higginson & Gilbert 2004; Schmickl & Crailsheim 2004; Schmickl, Thenius & Crailsheim 2012). Explicit calculations of foraging gain and costs are considered: for flying (Dukas & Edelstein‐Keshet 1998; Dornhaus *et al*. 2006a), for flying and walking (Higginson & Gilbert 2004), for two activity levels, depending also on the bees' weight (Schmickl & Crailsheim 2004; Schmickl, Thenius & Crailsheim 2012), for flying, depending on the bees' weight (Schmid‐Hempel, Kacelnik & Houston 1985), and for flying to, being at, and returning from a feeder (Johnson & Nieh 2010).

Most models use energetic efficiency as the currency that determines foraging decisions (Schmid‐Hempel, Kacelnik & Houston 1985; Dukas & Edelstein‐Keshet 1998; De Vries & Biesmeijer 2002; Higginson & Gilbert 2004; Schmickl & Crailsheim 2004; Dornhaus *et al*. 2006a; Johnson & Nieh 2010; Schmickl, Thenius & Crailsheim 2012). Some models contrast how well different currencies explain foragers' behaviour, that is, mainly the rate of energy influx (Schmid‐Hempel, Kacelnik & Houston 1985; Dukas & Edelstein‐Keshet 1998; Higginson & Gilbert 2004) or lifetime fitness (Dukas & Edelstein‐Keshet 1998). Higginson & Gilbert (2004) suggest a new currency, the energy profit per wingbeat linking the life span of foragers to the mechanical damage of flying.

## Utility of current models in understanding causes of colony mortality

Table 2 shows how the reviewed models could contribute to our current understanding of the factors contributing to colony failure, summarizing which models could be used to simulate the effect of each factor that has been implicated from empirical work (Neumann & Carreck 2010; Potts *et al*. 2010). Most of the models can only be used to assess the impact of one or two factors but combining several factors to address their interactions and their potential significance on honeybee decline is not explicitly considered by any of the published models. For some factors (impact of genetic diversity, bacterial pathogens, *Nosema* spp. and many beekeeping practices), no published model was found that could be directly used to simulate effects on colony survival.

**Table 2:**
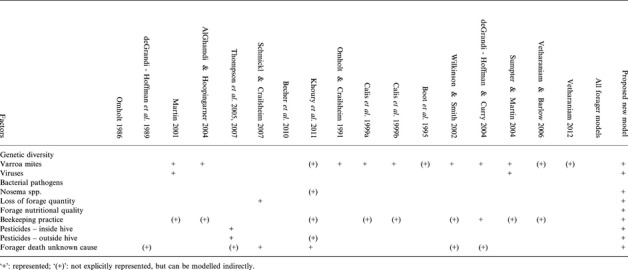
Factors potentially affecting the survival /death of a colony, and their representation in existing models

### Colony models

The contribution of most colony models to understanding honeybee decline is indirect by trying to capture the internal organization of a colony that is essential to any simulation of colony resilience. The main driver of colony dynamics is the queen's egg‐laying rate, but just imposing this rate is not sufficient because of the feedbacks between colony structure and egg laying: the number of nurse bees as well as the food influx will affect egg laying and brood survival and hence the future colony structure. The model that captures colony structure most comprehensively and has been most extensively tested is Schmickl & Crailsheim (2007); this gives the most flexibility for incorporating the effects of stressors acting within or outside the colony (other than pathogens).

### Varroa and pathogens

The model of Martin (2001) shows that it is important to describe the effect of varroa mites on honeybees in detail. It is not varroa infestation *per se* which drives a colony to extinction, but the viruses transmitted by varroa. Since different viruses have different effects on honeybee individuals and hence lead to different extinction dynamics, it seems important to take viruses into account explicitly, so the models incorporating varroa and virus dynamics (Martin 2001; Sumpter & Martin 2004) are considered most useful as building blocks of a future integrated model. A further insight from the varroa models is that timing of infestation and interaction with the honeybee cohort dynamics has a strong influence on varroa dynamics and chances of success of control measures. Hence, since honeybee cohort dynamics depends on weather, the current state of the colony and the incoming forage, it seems necessary to link varroa models to dynamic colony models rather than just imposing a static colony structure.

### Pesticides

Thompson *et al*. (2005, 2007) use a model to demonstrate that a sublethal effect induced by a pesticide, such as premature ageing, can be more detrimental to a colony than acute mortality over a short period of time. They used the size of the overwintering colony as an indicator of colony health and chances of survival the following year. Effects of changes in worker bee longevity can be counterintuitive, though, indicating a complex net of feedback mechanisms (Omholt 1986). Henry *et al*. (2012) use a model (Khoury, Myerscough & Barron 2011) to predict that sublethal effects of pesticides may lead to colony collapse although Cresswell & Thompson (2012) demonstrate that results change if spatiotemporal differences in colony growth during exposure are taken into account.

### Loss of forage or nutritional quality

None of the foraging models were related to colony survival or performance. Rather, these models focus on various aspects of collective central‐place foraging by a social insect (Fig. 1c). Whilst the varroa models are mostly driven by applied issues, that is, varroa control, the foraging models all focus on understanding more fundamental questions. Nevertheless, some important interfaces to colony structure have been explored in several models: the number of foraging and nectar‐receiving bees in the colony may be limited and lead to delays in foraging income and activity.

Foraging models were tested with simplified settings of one or two nectar sources in the landscape; several models used an experiment by Seeley, Camazine & Sneyd (1991) for verification. Interestingly, Schmickl & Crailsheim (2004) performed simulation experiments where they varied parameters of this experiment, leading to predictions that could be tested experimentally. Nevertheless, none of the foraging models considered foraging in realistic landscapes that are heterogeneous in time and space, and none of the models considered pollen foraging, let alone prioritization of pollen and nectar foraging.

Forager longevity (DeGrandi‐Hoffman *et al*. 1989) and mortality (Khoury, Myerscough & Barron 2011) can strongly affect colony size and survival, suggesting the need to couple colony and forager models to gain a better understanding of how different stressors interact and potentially result in colony decline and extinction. On the other hand, Schmickl & Crailsheim (2007) found that changes in parameters related to pollen foraging had no effects on colony dynamics, probably because existing feedback mechanisms were able to buffer changes in pollen foraging. Interestingly though, Khoury, Myerscough & Barron (2011) drove forager mortality to levels leading to extinction, whereas Schmickl & Crailsheim (2007) kept parameters within biologically plausible ranges.

## Building a new integrated model

Our review showed that many of the processes in honeybee colonies, epidemiology and foraging are well understood and well described in existing honeybee models. Thus, many building blocks for a comprehensive understanding of the resilience of honeybee colonies and their response to multiple stressors exist, but they have not yet been integrated in a cross‐level systems model. Below we (i) identify critical elements that could be used for future models; and (ii) outline the structure of a new, integrated ‘systems ecology’ model. We have used the same approach to develop our own integrated model, BEEHAVE (Becher *et al*., unpublished). It builds on existing submodels to provide a new tool that is more suitable for predicting the effects on environmental change on colonies than the reviewed models (which were not designed with this aim); (iii) We discuss benefits of this new integration to fill gaps identified in the models available to date. Additionally, a detailed discussion of the complexity, realism and importance of feedback loops is provided in Appendix S5 in Supporting Information.

### Building blocks

Our review shows that robust and tested conceptual designs of processes in existing models are suitable to be used as building blocks in an integrated model. A cohort‐based colony model using daily time steps seems to be the best basic design for a new integrated colony model. The model should include nectar and pollen consumption and stores so that linking it to an explicit foraging model makes sense.

With respect to the effects of pests and pathogens on bee colonies, integrated honeybee models can adopt more or less directly existing varroa models, but might need to include further elements to represent pathogens like *Nosema* spp. or pests like small hive beetle *Aethina tumida,* etc. The model by Martin (2001) is a good starting point as it already combines honeybee and varroa dynamics and includes the transmission of one of two viruses (DWV, APV) (being a combination of Martin (1998), DeGrandi‐Hoffman *et al*. (1989) and Boot *et al*. (1994, 1995)).

**Figure 2:**
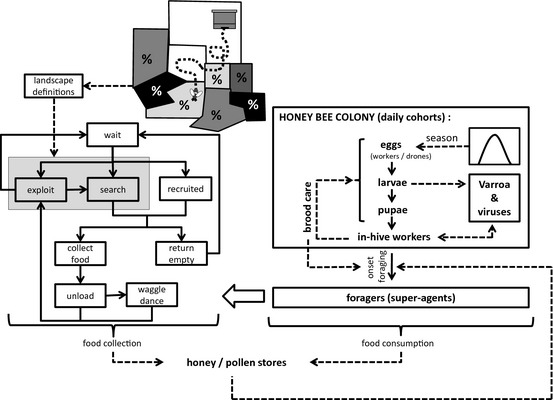
Simplified overview of the BEEHAVE model structure (Becher *et al*., unpublished): based on the egg‐laying rate and interacting with the varroa and foraging modules, the structure of a single honeybee colony is modelled. A separate landscape module allows to determine detection probabilities of flower patches (%) and to define their nectar and pollen flows over the season. This information is then taken into account, when foragers collect food in an agent‐based foraging module. Note that the various mortalities implemented in the model are not shown in this graph.

With respect to foraging behaviour, there are certain elements in published models which are useful to inform processes, although none of them simulates the effect of spatiotemporal variability of forage availability and quality on foraging success. Most of the foraging models distinguish between bees waiting in the hive, experienced foragers exploiting a known nectar source, dancers who recruit new bees to a profitable source and dance followers who will search for this specific source. Active foragers can abandon a patch and search another one or abandon foraging all together and rest. An integrated honeybee model should contain these basic activities of the foraging process to allow for realistic exploitation of food sources (Fig. 2). To assess the quality of a nectar source, usually energetic efficiency is preferred as a currency, but a model offering a choice of alternative currencies would be more flexible.

### Framework for a new, integrated model

In Fig. 2, we present a concept of how an integrated model could be designed, using structures identified above, but also incorporating processes and feedbacks that are not included in published models. The benefits of its modular design would be that modules can be run and tested independently of each other. Additionally, complexity is cut down, as only certain data are exchanged between the modules, for example, the foraging module would get the current foraging force as input and provide changes in nectar stores, pollen stores and number of foragers as output.

Further factors that affect colony survival (Table 2) can be added relatively easily, especially if they only require adjustments of parameter values. For example, effects of pesticides inside the hive could be simulated by increasing the brood mortality and effects of pesticide exposure outside the hive by increasing the foraging mortality. New varroa‐transmitted viruses could be defined by modified transmission and mortality rates. Nosemoses may be represented by an increased in‐hive mortality rate and higher food consumption rates of infected bees.

The design of our own upcoming integrated model BEEHAVE will be based on the framework described above (Fig. 2). It can be used to examine the effects of more stressors than other models (as shown in the last column of Table 2). As a conceptual example of its use to study interactions between stressors, we can simulate the effect of different populations of varroa mites on colony survival in landscapes with differing floral availability. As the colony and foraging module affect each other via the income of food, expenditure of energy and the differing mortalities within and outside the hive, then it becomes apparent that the effect of varroa mites on colony survival is actually modulated by the energetic efficiency of different nectar sources in the landscape.

### Benefits of new framework compared with reviewed models

#### Coupling of colony dynamics and foraging

One key finding of the review is that no one has tried to link a dynamic colony model with a dynamic foraging model. Those colony models that include a short‐term feedback from colony structure on foraging do this by making the number of foragers depend on colony structure and, in one case (Schmickl & Crailsheim 2007), on stores of pollen and nectar (Table S4). The number of foragers in turn influences colony structure indirectly, via incoming forage. In BEEPOP (DeGrandi‐Hoffman *et al*. 1989), for example, the queen's egg‐laying rate is affected by the number of foragers. It can be assumed, though, that short‐term changes in forage availability (e.g. due to weather) also affect within‐hive processes. Linking colony and foraging processes dynamically is explicit in the new framework.

#### Pollen collection

Existing foraging models focus on nectar collection but neglect pollen and only in one colony model, HoPoMo, is pollen consumption considered. The availability of pollen and protein‐rich jelly from nurse bees is essential for raising larvae and a lack of pollen supply will severely affect the colony growth, so pollen collection has been added to the integrated model framework.

#### Foraging in heterogeneous, dynamic landscapes

None of the foraging models were linked to a representation of real landscapes, characterized by mosaic of patches like arable fields or orchards providing nectar and pollen for a certain amount of time. This makes it impossible to explore the potential contribution of changes in land use and agricultural practice to colony losses. Some of these aspects (use of land cover data, floral resources, foraging distances) are implemented in a model by Lonsdorf *et al*. (2009). Although we did not include it into our review as it is a general pollinator model with the focus on pollination services, it would be of use for developing the landscape module of an integrated model.

#### Pesticides

Only three models explore effects of pesticides on a colony (Thompson *et al*. 2005, 2007; Cresswell & Thompson 2012; Henry *et al*. 2012), which are very simple and were originally designed for other purposes. These models seem to be too simple to represent all important resilience mechanisms of colony and their capacity. Nevertheless, these models clearly demonstrate the potential significance of pesticides, in particular of sublethal effects that were found to impose a much larger risk than acute effects (Thompson *et al*. 2005, 2007).

It is important to test the effects of pesticides in comprehensive models that can take complex exposure landscapes into account (Osborne 2012), and this is feasible with the structure shown in Fig 2. One other such attempt exists: BEEPOP has been augmented by detailed modules for including effects of pesticides, implemented in the software PC BEEPOP (Bromenshenk *et al*. 1991). The module BEETOX includes a toxicity data base for more than 400 chemicals and calculated lethal and sublethal effects for specific exposures; the module BEEKILL allowed the user to link these effects to exposure scenarios and feed the resulting changes in mortality, development and longevity into the colony model. Unfortunately, details of these modules were not published and it seems that it has never been used for regulatory risk assessment of pesticides.

#### Models on the effects of other stressors

We did not find published mechanistic models predicting the impact of bacterial or microsporidian (*Nosema* spp.) infection on a honeybee colony. Likewise, there were no models calculating the risks of limited genetic diversity on colony growth or the impact of many beekeeping practices, apart from some varroa treatments. Some of these potential stressors might be easily added to the suggested integrated model, changes in parameter values (e.g. mortality rates, foraging probabilities etc.). However, if they require substantial structural adaptions and interactions of different parts of the model, then it would be necessary to develop a specific new module.

#### Honeybee population dynamics

Our proposed framework only focuses on a single colony. To model honeybee population dynamics, all colonies (including both wild and managed) in a sufficiently large area would need to be represented and swarming, robbing, drifting and the availability of suitable natural nest sites would need to be incorporated. Of the reviewed models, only HoPoMo represents colony division, but no landscape‐level population dynamics were simulated. Neighbouring colonies as a source of continuous mite invasion are taken into account by Calis, Fries & Ryrie (1999b) and Vetharaniam & Barlow (2006). Current population models either keep the within‐hive colony dynamics extremely simple (e.g. Al‐Khafaji *et al*. 2009) or neglect it completely (e.g. Matis & Kiffe 2002; Mistro, Rodrigues & Ferreira 2005). To extrapolate to population models, including the interaction among different colonies, the strategy used in modelling metapopulations will be useful.

### Conclusion and application

First, this review provides a detailed report of published honeybee models that can be used by scientists to decide which published model might be suitable for their specific needs. Many processes within colonies, epidemiology and foraging are well understood and described in the models, and there are multiple feedback loops that regulate colony dynamics and can buffer the colony against changes in the environment. However, recent colony losses suggest this resilience may not be powerful enough to withstand multiple pressures over time and space; we found no published model that coupled in‐hive colony dynamics with pathology and with foraging dynamics of bees in heterogeneous landscapes. Since such an understanding is essential to the continued management of bee colonies, we have therefore proposed a structure for a new integrated model, building on those already available, to capture the impact of stressors affecting bees within the hive (such as disease and management factors) together with the impact of factors affecting bees whilst foraging (such as floral availability, weather or pesticide exposure). The development of such a model that can predict the survival and productivity of honeybee colonies under different scenarios enables us to highlight when ‘tipping points’ are likely to be reached with different combinations of factors and show which hypotheses should be prioritized for empirical testing. Importantly, such a systems model could also be utilized by policy makers, land managers and beekeepers to forecast the effects of environmental change and implementation of different management options. Three examples are given if such a modelling tool was made readily available to a diversity of stakeholders. (i) A beekeeper could use the model to predict the effects of different apiary locations, with contrasting forage availability on colony growth and survival, under realistic assumptions of colony size, varroa load and management regime; (ii) Regulatory authorities and agro‐chemical companies could utilize the model to better evaluate complex pesticide exposure landscapes and the likely effect of exposure on individual bees and colony level responses. (iii) The relative benefits of different areas and locations of sown forage mixtures on the survival of large and small colonies (for example) could be examined in silico. In turn, this would ensure that advice given for agri‐environment schemes and resources spent on planting such mixtures are more likely to result in benefits to the local colonies.

## Supporting information


**Table S1.** Papers with honeybee models considered and included or not included in this review.

**Table S2.** Additional information on the models listed in Table 1: Model output presented (all models), honeybee representation (varroa models), recruitment (foraging models).

**Table S3.** Listing entities and state variables of the reviewed colony models.

**Table S4.** Listing processes and feedbacks of the reviewed colony models.

**Appendix S5.** Discussion of the complexity, and importance of feedback loops of the reviewed models
