# Supplementary material for: REVIEW: Towards a systems approach for understanding honeybee decline: a stocktaking and synthesis of existing models
Source: J Appl Ecol. 2013 Jun 10;50(4):868–80. doi: 10.1111/1365-2664.12112 (PMC3810709; doi:10.1111/1365-2664.12112)
Supplement: Supplementary file 1 — Table S1. Papers with honeybee models considered and included or not included in this review. [file JPE-50-868-s001.pdf]

Supporting Information: Becher et al: Modelling Honeybee colonies

S1a: Colony and Varroa models

Authors	Year	reviewed	Comment	Full reference
Al-Khafaji, K., S. Tuljapurkar, et al.	2009		Focus on population dynamics of colonies; single colonies described using the logistic equation.	Al-Khafaji, K., S. Tuljapurkar, et al. (2009). Hierarchical demography: a general approach with an application to honey bees. Ecology 90(2): 556-566.
Amdam, G. V. and S. C. Seehuus	2006		No model.	Amdam, G. V. and S. C. Seehuus (2006). Order, disorder, death: Lessons from a superorganism. Advances in Cancer Research, Vol 95 G. F. VandeWoude and G. Klein. 95:00:00 31-60.
Antolin, M. F.	1999		Different species: parasitoid wasps	Antolin, M. F. (1999). A genetic perspective on mating systems and sex ratios of parasitoid wasps. Researches on Population Ecology 41(1): 29-37.
Beauchamp, G.	1992		Focus on optimal foraging, # brood and in-hive bees assumed to be constant, only 20 days at end of foraging season considered.	Beauchamp, G. (1992). EFFECTS OF ENERGY-REQUIREMENTS AND WORKER MORTALITY ON COLONY GROWTH AND FORAGING IN THE HONEY-BEE. Behavioral Ecology and Sociobiology 31(2): 123-132.
Becher, M. A., H. Hildenbrandt, et al.	2010	yes		Becher, M. A., H. Hildenbrandt, et al. (2010). Brood temperature, task division and colony survival in honeybees: A model. Ecological Modelling 221(5): 769-776.
Boot, W. J., M. Vanbaalen, et al.	1995	yes		Boot, W. J., M. Vanbaalen, et al. (1995). WHY DO VARROA MITES INVADE WORKER BROOD CELLS OF THE HONEY-BEE DESPITE LOWER REPRODUCTIVE SUCCESS. Behavioral Ecology and Sociobiology 36(4): 283-289.
Bowen-Walker, P. L. and A. Gunn	1998		No model.	Bowen-Walker, P. L. and A. Gunn (1998). Inter-host transfer and survival of Varroa jacobsoni under simulated and natural winter conditions. Journal of Apicultural Research 37(3): 199-204.
Branco, M. R., N. A. C. Kidd, et al.	1999		Field study and statistical model.	Branco, M. R., N. A. C. Kidd, et al. (1999). Development of Varroa jacobsoni in colonies of Apis mellifera iberica in a Mediterranean climate. Apidologie 30(6): 491-503.
Brodsgaard, C. J., H. Hansen, et al.	2000		No model.	Brodsgaard, C. J., H. Hansen, et al. (2000). Progress of Paenibacillus larvae larvae infection in individually inoculated honey bee larvae reared singly in vitro, in micro colonies, or in full-size colonies. Journal of Apicultural Research 39(1-2): 19-27.
Bromenshenk, J. J., J. Doskocil, et al.	1991		Only description of software, no model description included.	Bromenshenk, J. J., J. Doskocil, et al. (1991). PC BEEPOP, AN ECOTOXICOLOGICAL SIMULATION-MODEL FOR HONEY-BEE POPULATIONS. Environmental Toxicology and Chemistry 10(4): 547-558.

Authors	Year	reviewed	Comment	Full reference
Brown, M. J. F. and P. Schmid-Hempel	2003		Different subject.	Brown, M. J. F. and P. Schmid-Hempel (2003). The evolution of female multiple mating in social hymenoptera. Evolution 57(9): 2067-2081.
Calatayud, F. and M. J. Verdu	1995		Field study and statistical model.	Calatayud, F. and M. J. Verdu (1995). NUMBER OF ADULT FEMALE MITES VARROA-JACOBSONI OUD ON HIVE DEBRIS FROM HONEY-BEE COLONIES ARTIFICIALLY INFESTED TO MONITOR MITE POPULATION INCREASE (MESOSTIGMATA, VARROIDAE). Experimental & Applied Acarology 19(3): 181-188.
Calis, J. N. M., W. J. Boot, et al.	1999	yes		Calis, J. N. M., W. J. Boot, et al. (1999). Model evaluation of methods for Varroa jacobsoni mite control based on trapping in honey bee brood. Apidologie 30(2-3): 197-207.
Calis, J. N. M., W. J. Boot, et al.	1999		No model.	Calis, J. N. M., W. J. Boot, et al. (1999). Effective biotechnical control of varroa: applying knowledge on brood cell invasion to trap honey bee parasites in drone brood. Journal of Apicultural Research 38(1-2): 49-61.
Calis, J. N. M., I. Fries, et al.	1999	yes		Calis, J. N. M., I. Fries, et al. (1999). Population modelling of Varroa jacobsoni Oud. Apidologie 30(2-3): 111-124.
Cook, D. W. M.	2002		Different species: Fungi	Cook, D. W. M. (2002). A laboratory simulation for vectoring of Trichosporon pullulans by conidia of Botrytis cinerea. Phytopathology 92(12): 1293-1299.
Davidson, G., K. Phelps, et al.	2003		Different subject.	Davidson, G., K. Phelps, et al. (2003). Study of temperature-growth interactions of entomopathogenic fungi with potential for control of Varroa destructor (Acari : Mesostigmata) using a nonlinear model of poikilotherm development. Journal of Applied Microbiology 94(5): 816-825.
Degrandihoffman, G., S. A. Roth, et al.	1989	yes		Degrandihoffman, G., S. A. Roth, et al. (1989). BEEPOP - A HONEYBEE POPULATION-DYNAMICS SIMULATION-MODEL. Ecological Modelling 45(2): 133-150.
DeGrandi-Hoffman, G. and R. Curry	2004	yes		DeGrandi-Hoffman, G. and R. Curry (2004). A mathematical model of varroa mite (Varroa destructor Anderson and Trueman) and honeybee (Apis mellifera L.) population dynamics. International Journal of Acarology 30(3): 259-274.
Degrandi-Hoffman, G. and R. Curry	2005	(yes)	based on DeGrandi-Hoffman and Curry 2004	Degrandi-Hoffman, G. and R. Curry (2005). Simulated population dynamics of Varroa Mites in honey bee colonies: Part II - What the VARROAPOP model reveals. American Bee Journal 145(8): 629-632.
Deneubourg, J. L., A. Lioni, et al.	2002		Different subject.	Deneubourg, J. L., A. Lioni, et al. (2002). Dynamics of aggregation and emergence of cooperation. Biological Bulletin 202(3): 262-267.
Fahrbach, S. E., S. M. Farris, et al.	2003		Different subject.	Fahrbach, S. E., S. M. Farris, et al. (2003). Limits on volume changes in the mushroom bodies of the honey bee brain. Journal of Neurobiology 57(2): 141-151.
Fewell, J. H. and S. M. Bertram	1999		Field study to test models on division of labour	Fewell, J. H. and S. M. Bertram (1999). Division of labor in a dynamic environment: response by honeybees (Apis mellifera) to graded changes in colony pollen stores. Behavioral Ecology and Sociobiology 46(3): 171-179.
Frank, S. A.	1997		Different subject.	Frank, S. A. (1997). The design of adaptive systems: Optimal parameters for variation and selection in learning and development. Journal of Theoretical Biology 184(1): 31-39.

S1a: Colony and Varroa models

Authors	Year	reviewed	Comment	Full reference
Fries, I., S. Camazine, et al.	1994	yes		Fries, I., S. Camazine, et al. (1994). POPULATION-DYNAMICS OF VARROA-JACOBSONI - A MODEL AND A REVIEW. Bee World 75(1): 5-28.
Fries, I. and S. Perez-Escala	2001		No model.	Fries, I. and S. Perez-Escala (2001). Mortality of Varroa destructor in honey bee (Apis mellifera) colonies during winter. Apidologie 32(3): 223-229.
Hayter, K. E. and J. E. Cresswell	2006		Field study on pollination	Hayter, K. E. and J. E. Cresswell (2006). The influence of pollinator abundance on the dynamics and efficiency of pollination in agricultural Brassica napus: implications for landscape-scale gene dispersal. Journal of Applied Ecology 43(6): 1196-1202.
Jarne, P. and P. J. L. Lagoda	1996		Different subject.	Jarne, P. and P. J. L. Lagoda (1996). Microsatellites, from molecules to populations and back. Trends in Ecology & Evolution 11(10): 424-429.
Johnson, B. R.	2009		Different subject: Pattern formation on combs	Johnson, B. R. (2009). Pattern formation on the combs of honeybees: increasing fitness by coupling self-organization with templates. Proceedings of the Royal Society B-Biological Sciences 276(1655): 255-261.
Johnson, B. R.	2010		Different subject: division of Labour	Johnson, B. R. (2010). Division of labor in honeybees: form, function, and proximate mechanisms. Behavioral Ecology and Sociobiology 64(3): 305-316.
Karsai, I. and T. Schmickl	2011		Different subject: comb construction in wasps	Karsai, I. and T. Schmickl (2011). Regulation of task partitioning by a common stomach": a model of nest construction in social wasps." Behavioral Ecology 22(4): 819-830.
Kasper, M. L., A. F. Reeson, et al.	2008		Different species: Vespa	Kasper, M. L., A. F. Reeson, et al. (2008). Environmental factors influencing daily foraging activity of Vespula germanica (Hymenoptera, Vespidae) in Mediterranean Australia. Insectes Sociaux 55(3): 288-295.
Khoury, D. S., M. R. Myerscough, et al.	2011	yes		Khoury, D. S., M. R. Myerscough, et al. (2011). A Quantitative Model of Honey Bee Colony Population Dynamics. Plos One 6(4).
Kleineidam, C. and F. Roces	2000		No model, different species.	Kleineidam, C. and F. Roces (2000). Carbon dioxide concentrations and nest ventilation in nests of the leaf-cutting ant Atta vollenweideri. Insectes Sociaux 47(3): 241-248.
Kudo, K., S. Koji, et al.	2011		Different species: neotropical wasps	Kudo, K., S. Koji, et al. (2011). Worker demography in a large-colony, swarm-founding wasp. Population Ecology 53(2): 297-306.
Maggi, M., N. Damiani, et al.	2010		Field study and statistical model.	Maggi, M., N. Damiani, et al. (2010). Brood cell size of Apis mellifera modifies the reproductive behavior of Varroa destructor. Experimental and Applied Acarology 50(3): 269-279.
Makela, M. E., G. A. Rowell, et al.	1993		Model description incomplete and unavailable; very little evidence of model testing and analysis.	Makela, M. E., G. A. Rowell, et al. (1993). AN OBJECT-ORIENTED INTRACOLONIAL AND POPULATION-LEVEL MODEL OF HONEY-BEES BASED ON BEHAVIORS OF EUROPEAN AND AFRICANIZED SUBSPECIES. Ecological Modelling 67(2-4): 259-284.

Authors	Year	reviewed	Comment	Full reference
Maleszka, J., A. B. Barron, et al.	2009		Different subject.	Maleszka, J., A. B. Barron, et al. (2009). Effect of age, behaviour and social environment on honey bee brain plasticity. Journal of Comparative Physiology a-Neuroethology Sensory Neural and Behavioral Physiology 195(8): 733-740.
Martin, S.	1998	yes		Martin, S. (1998). A population model for the ectoparasitic mite Varroa jacobsoni in honey bee (Apis mellifera) colonies. Ecological Modelling 109(3): 267-281.
Martin, S., T. Wossler, et al.	2002		Different species.	Martin, S., T. Wossler, et al. (2002). Usurpation of African Apis mellifera scutellata colonies by parasitic Apis mellifera capensis workers. Apidologie 33(2): 215-231.
Martin, S. J.	2001	yes		Martin, S. J. (2001). The role of Varroa and viral pathogens in the collapse of honeybee colonies: a modelling approach. Journal of Applied Ecology 38(5): 1082-1093.
Matis, J. H. and T. R. Kiffe	2002		Different subspecies (African honeybee), focus on mathematical representation	Matis, J. H. and T. R. Kiffe (2002). On interacting bee/mite populations: a stochastic model with analysis using cumulant truncation. Environmental and Ecological Statistics 9(3): 237-258.
Matis, J. H., Q. Zheng, et al.	1995		Different subspecies (African honeybee), focus on mathematical representation	Matis, J. H., Q. Zheng, et al. (1995). DESCRIBING THE SPREAD OF BIOLOGICAL POPULATIONS USING STOCHASTIC COMPARTMENTAL-MODELS WITH BIRTHS. Mathematical Biosciences 126(2): 215-247.
Meikle, W. G., J. M. Patt, et al.	2012		Field study on small hive beetle	Meikle, W. G., J. M. Patt, et al. (2012). Intraspecific Competition Effects on Aethina tumida (Coleoptera: Nitidulidae). Journal of Economic Entomology 105(1): 26-33.
Meikle, W. G., B. G. Rector, et al.	2008		no model	Meikle, W. G., B. G. Rector, et al. (2008). Within-day variation in continuous hive weight data as a measure of honey bee colony activity. Apidologie 39(6): 694-707.
Mistro, D. C., L. A. D. Rodrigues, et al.	2005		Different species.	Mistro, D. C., L. A. D. Rodrigues, et al. (2005). The Africanized honey bee dispersal: a mathematical zoom. Bulletin of Mathematical Biology 67(2): 281-312.
Mondragon, L., M. Spivak, et al.	2005		Statistical model	Mondragon, L., M. Spivak, et al. (2005). A multifactorial study of the resistance of honeybees Apis mellifera to the mite Varroa destructor over one year in Mexico. Apidologie 36(3): 345-358.
Moretto, G.	2002		No model.	Moretto, G. (2002). Mortality of Varroa destructor in broodless africanized and carnica honey bee (Apis mellifera L.) colonies. Interciencia 27(12): 702-704.
Moritz, R. F. A.	2002		Different subspecies (Cape honey bee).	Moritz, R. F. A. (2002). Population dynamics of the Cape bee phenomenon: The impact of parasitic laying worker clones in apiaries and natural populations. Apidologie 33(2): 233-244.
Murilhas, A. M.	2002		No model.	Murilhas, A. M. (2002). Varroa destructor infestation impact on Apis mellifera carnica capped worker brood production, bee population and honey storage in a Mediterranean climate. Apidologie 33(3): 271-281.

Authors	Year	reviewed	Comment	Full reference
Naug, D. and S. Camazine	2002		Behavioural time scale	Naug, D. and S. Camazine (2002). The role of colony organization on pathogen transmission in social insects. Journal of Theoretical Biology 215(4): 427-439.
O'Donnell, S. and S. J. Bulova	2007		Different subject: division of Labour.	O'Donnell, S. and S. J. Bulova (2007). Worker connectivity: a simulation model of variation in worker communication and its effects on task performance. Insectes Sociaux 54(3): 211-218.
Ohtsuki, H. and K. Tsuji	2009		Different subject: Game model on evolution of worker policing	Ohtsuki, H. and K. Tsuji (2009). Adaptive Reproduction Schedule as a Cause of Worker Policing in Social Hymenoptera: A Dynamic Game Analysis. American Naturalist 173(6): 747-758.
Oliver, R.	2007		based on existing models, e.g. DeGrandi-Hoffmans Varroapop	Oliver, R. (2007). Fighting varroa: The silver bullet, or brass knuckles? - Strategy and tactics - Third in a series on integrated pest management of varroa. American Bee Journal 147(2): 135-140.
Oliver, R.	2011		tool for beekeepers, no description of the actual model	Oliver, R. (2011). SICK BEES PART 12 Varroa Management-Getting Down to the Brass Tacks. American Bee Journal 151(9): 859-866.
Planque, R., J. B. van den Berg, et al.	2010		Different species (ants)	Planque, R., J. B. van den Berg, et al. (2010). Recruitment Strategies and Colony Size in Ants. Plos One 5(8).
Pratt, S. C.	1999		Different subject.	Pratt, S. C. (1999). Optimal timing of comb construction by honeybee (Apis mellifera) colonies: a dynamic programming model and experimental tests. Behavioral Ecology and Sociobiology 46(1): 30-42.
Pratt, S. C., D. J. T. Sumpter, et al.	2005		Different species: Temnothorax (ant)	Pratt, S. C., D. J. T. Sumpter, et al. (2005). An agent-based model of collective nest choice by the ant Temnothorax albipennis. Animal Behaviour 70:00:00 1023-1036.
Quijano, N. and K. M. Passino	2010		focus on "bio-inspired" algorithms for to solve engineering problems	Quijano, N. and K. M. Passino (2010). Honey bee social foraging algorithms for resource allocation: Theory and application. Engineering Applications of Artificial Intelligence 23(6): 845-861.
Reich, S. E., S. Fuchs, et al.	1998		Different subject.	Reich, S. E., S. Fuchs, et al. (1998). Geometric approximation of the infestation of honey bee brood cells by Varroa jacobsoni and implications for the estimation of brood infestation, for population models and for the proportion of non-sibling matings. Journal of Apicultural Research 37(2): 115-123.
Rinderer, T. E., L. I. De Guzman, et al.	2001		Field study	Rinderer, T. E., L. I. De Guzman, et al. (2001). Multi-state field trials of ARS Russian honey bees - 1. Responses to Varroa destructor 1999, 2000. American Bee Journal 141(9): 658-661.
Roubik, D. W. and H. Wolda	2001		No model.	Roubik, D. W. and H. Wolda (2001). Do competing honey bees matter? Dynamics and abundance of native bees before and after honey bee invasion. Population Ecology 43(1): 53-62.

S1a: Colony and Varroa models

Authors	Year	reviewed	Comment	Full reference
Royce, L. A., B. A. Stringer, et al.	1993		Experimental study	Royce, L. A., B. A. Stringer, et al. (1993). INFESTATION OF TRACHEAL MITES (ACARI, TARSONEMIDAE) IN FERAL AND MANAGED COLONIES OF HONEY-BEES (HYMENOPTERA, APIDAE). Journal of Economic Entomology 86(3): 712-714.
Ruiz-Gonzalez, M. X. and M. J. F. Brown	2006		Field study	Ruiz-Gonzalez, M. X. and M. J. F. Brown (2006). Honey bee and bumblebee trypanosomatids: specificity and potential for transmission. Ecological Entomology 31(6): 616-622.
Schmickl, T. and K. Crailsheim	2007	yes		Schmickl, T. and K. Crailsheim (2007). HoPoMo: A model of honeybee intracolonial population dynamics and resource management. Ecological Modelling 204(1-2): 219-245.
Stalidzans, E., Z. Markovitch, et al.	2001		Conference abstract	Stalidzans, E., Z. Markovitch, et al. (2001). Simulation of death dynamics of wintering generation of bees under different climatical conditions.
Stokes, K. E., Y. M. Buckley, et al.	2006		Different species: Bombus	Stokes, K. E., Y. M. Buckley, et al. (2006). A modelling approach to estimate the effect of exotic pollinators on exotic weed population dynamics: bumblebees and broom in Australia. Diversity and Distributions 12(5): 593-600.
Sumpter, D. J. T. and D. S. Broomhead	2001		Different species: A. cerana	Sumpter, D. J. T. and D. S. Broomhead (2001). Relating individual behaviour to population dynamics. Proceedings of the Royal Society of London Series B-Biological Sciences 268(1470): 925-932.
Sumpter, D. J. T. and S. J. Martin	2004	yes		Sumpter, D. J. T. and S. J. Martin (2004). The dynamics of virus epidemics in Varroa-infested honey bee colonies. Journal of Animal Ecology 73(1): 51-63.
Tarapore, D., D. Floreano, et al.	2010		Different subject: division of Labour.	Tarapore, D., D. Floreano, et al. (2010). Task-dependent influence of genetic architecture and mating frequency on division of labour in social insect societies. Behavioral Ecology and Sociobiology 64(4): 675-684.
Thierry, B., G. Theraulaz, et al.	1995		Different subject: joint memory	Thierry, B., G. Theraulaz, et al. (1995). Joint memory. Behavioural Processes 35(1-3): 127-140.
Thompson, H. M.	2003		No model.	Thompson, H. M. (2003). Behavioural effects of pesticides in bees - Their potential for use in risk assessment. Ecotoxicology 12(1-4): 317-330.
Thompson, H. M., S. Wilkins, et al.	2005	yes		Thompson, H. M., S. Wilkins, et al. (2005). The effects of four insect growth-regulating (IGR) insecticides on honeybee (Apis mellifera l.) colony development, queen rearing and drone sperm production. Ecotoxicology 14(7): 757-769.
Thompson, H. M., S. Wilkins, et al.	2007	yes		Thompson, H. M., S. Wilkins, et al. (2007). Modelling long-term effects of IGRs on honey bee colonies. Pest Management Science 63(11): 1081-1084.
Vetharaniam, I.	2012	yes		Vetharaniam, I. (2012). Predicting reproduction rate of varroa. Ecological Modelling 224(1): 11-17.
Vetharaniam, I. and N. D. Barlow	2006	yes		Vetharaniam, I. and N. D. Barlow (2006). Modelling biocontrol of Varroa destructor using a benign haplotype as a competitive antagonist. New Zealand Journal of Ecology 30(1): 87-102.

Supporting Information: Becher et al: Modelling Honeybee colonies

S1a: Colony and Varroa models

Authors	Year	reviewed	Comment	Full reference
Waters, J. S., C. T. Holbrook, et al.	2010		Different species: Pogonomyrmex (ant)	Waters, J. S., C. T. Holbrook, et al. (2010). Allometric Scaling of Metabolism, Growth, and Activity in Whole Colonies of the Seed-Harvester Ant Pogonomyrmex californicus. American Naturalist 176(4): 501-510.
Wilkinson, D. and G. C. Smith	2002	yes		Wilkinson, D. and G. C. Smith (2002). A model of the mite parasite, Varroa destructor, on honeybees (Apis mellifera) to investigate parameters important to mite population growth. Ecological Modelling 148(3): 263-275.
Yanez, O., R. Jaffe, et al.	2012		Field study	Yanez, O., R. Jaffe, et al. (2012). Deformed wing virus and drone mating flights in the honey bee (Apis mellifera): implications for sexual transmission of a major honey bee virus. Apidologie 43(1): 17-30.
Zhao, X., V. Coptis, et al.	2008		Different species: Tribolium (beetle)	Zhao, X., V. Coptis, et al. (2008). Metamorphosis and Adult Development of the Mushroom Bodies of the Red Flour Beetle, Tribolium castaneum. Developmental Neurobiology 68(13): 1487-1502.
Al Ghamdi and Hoopingarner	2004	yes	Additional	Al Ghamdi, A. & Hoopingarner R. (2004) Modeling of honey bee and varroa mite population dynamics. *Saudi Journal of biological sciences* . 11, 21-36.
Omholt	1986	yes	Additional	Omholt, S. (1986) A model for intracolonial population dynamics of the honeybee in temperate zones. *Journal of Apicultural Research* , 25:9–21.
Omholt & Crailsheim	1991	yes	Additional	Omholt, S.W. & Crailsheim, K. (1991) The possible prediction of the degree infestation of honeybee colonies (*Apis mellifera*) by *Varroa jacobsoni* OUD. by means of its natural death-rate: a dynamic model approach. *Norwegian Journal of Agricutural Sciences* , 5, 393-400.

S1b: Foraging models

Authors	Year	reviewed	Comment	Full reference
Burns and Thomson	2006		Different species: bumble bees	J. G. Burns and J. D. Thomson. A test of spatial memory and movement patterns of bumblebees at multiple spatial and temporal scales. *Behavioral Ecology* 17 (1):48-55, 2006.
Cresswell	1997		Different species: bumble bees	J. E. Cresswell. Spatial heterogeneity, pollinator behaviour and pollinator-mediated gene flow: Bumblebee movements in variously aggregated rows of oil-seed rape. *Oikos* 78 (3):546-556, 1997.
Cresswell et al.	1995		Focus on pollen distribution	J. E. Cresswell, A. P. Bassom, S. A. Bell, S. J. Collins, and T. B. Kelly. Predicted pollen dispersal by honey-bees and three species of bumble-bees foraging on oil-seed rape: A comparison of three models. *Functional Ecology* 9 (6):829-841, 1995.
Cresswell et al.	2000		Different species: bumble bees	J. E. Cresswell, J. L. Osborne, and D. Goulson. An economic model of the limits to foraging range in central place foragers with numerical solutions for bumblebees. *Ecological Entomology* 25 (3):249-255, 2000.
Diekotter et al.	2006		Different species: bumble bees	T. Diekotter, K. Walther-Hellwig, M. Conradi, M. Suter, and R. Frankl. Effects of landscape elements on the distribution of the rare bumblebee species Bombus muscorum in an agricultural landscape. *Biodiversity and Conservation* 15 (1):57-68, 2006.
Dreisig	1997		Different species: bumble bees	H. Dreisig. Why do some nectar foragers perch and others hover while probing flowers? *Evolutionary Ecology* 11 (5):543-555, 1997.
Dukas and Edelstein-Keshet	1998	yes		R. Dukas and L. Edelstein-Keshet. The spatial distribution of colonial food provisioners. *Journal of Theoretical Biology* 190 (2):121-134, 1998.
Higginson and Gilbert	2004	yes		A. D. Higginson and F. Gilbert. Paying for nectar with wingbeats: a new model of honeybee foraging. *Proceedings of the Royal Society of London Series B-Biological Sciences* 271 (1557):2595-2603, 2004.
Jordan and Harder	2006		Focus on plant architecture	C. Y. Jordan and L. D. Harder. Manipulation of bee behavior by inflorescence architecture and its consequences for plant mating. *American Naturalist* 167 (4):496-509, 2006.
Mitesser et al.	2006		Different species: halictid bees	O. Mitesser, N. Weissel, E. Strohm, and H. J. Poethke. The evolution of activity breaks in the nest cycle of annual eusocial bees: a model of delayed exponential growth. *Bmc Evolutionary Biology* 6, 2006.
Ohashi and Thomson	2009		Different species: bumble bees	K. Ohashi and J. D. Thomson. Trapline foraging by pollinators: its ontogeny, economics and possible consequences for plants. *Annals of Botany* 103 (9):1365-1378, 2009.
Ohashi and Yahara	2002		Different species: bumble bees	K. Ohashi and T. Yahara. Visit larger displays but probe proportionally fewer flowers: counterintuitive behaviour of nectar-collecting bumble bees achieves an ideal free distribution. *Functional Ecology* 16 (4):492-503, 2002.
Osborne et al.	2008		Different species: bumble bees	J. L. Osborne, A. P. Martin, N. L. Carreck, J. L. Swain, M. E. Knight, D. Goulson, R. J. Hale, and R. A. Sanderson. Bumblebee flight distances in relation to the forage landscape. *Journal of Animal Ecology* 77 (2):406-415, 2008.
Petrikin and Wells	1995		Experimental study	J. Petrikin and H. Wells. Honey bee (Apis mellifera) use of flower pigment patterns in making foraging choices. *Journal of the Kansas Entomological Society* 68 (4):377-387, 1995.
Pratt	1999		Focus on comb construction	S. C. Pratt. Optimal timing of comb construction by honeybee (Apis mellifera) colonies: a dynamic programming model and experimental tests. *Behavioral Ecology and Sociobiology* 46 (1):30-42, 1999.
Raine et al.	2009		Different species: bumble bees	N. E. Raine, D. K. Rossmo, and S. C. Le Comber. Geographic profiling applied to testing models of bumble-bee foraging. *Journal of the Royal Society Interface* 6 (32):307-319, 2009.

S1b: Foraging models

Authors	Year	reviewed	Comment	Full reference
Rands and Whitney	2008		Different species: bumble bees	S. A. Rands and H. M. Whitney. Floral Temperature and Optimal Foraging: Is Heat a Feasible Floral Reward for Pollinators? *Plos One* 3 (4), 2008.
Real	1991		Different species: bumble bees	L. A. Real. Animal Choice Behavior and the Evolution of Cognitive Architecture. *Science* 253 (5023):980-986, 1991.
Roulston and Silverman	2002		Different species: ants	T. H. Roulston and J. Silverman. The effect of food size and dispersion pattern on retrieval rate by the Argentine ant, Linepithema humile (Hymenoptera : formicidae). *Journal of Insect Behavior* 15 (5):633-648, 2002.
Spaethe et al.	2001		Different species: bumble bees	J. Spaethe, J. Tautz, and L. Chittka. Visual constraints in foraging bumblebees: Flower size and color affect search time and flight behavior. *Proceedings of the National Academy of Sciences of the United States of America* 98 (7):3898-3903, 2001.
Stout and Goulson	2002		Different species: bumble bees	J. C. Stout and D. Goulson. The influence of nectar secretion rates on the responses of bumblebees (Bombus spp.) to previously visited flowers. *Behavioral Ecology and Sociobiology* 52 (3):239-246, 2002.
Strickler	1996		Different species: solitary bees	K. Strickler. Seed and bee yields as a function of forager populations: Alfalfa pollination as a model system. *Journal of the Kansas Entomological Society* 69 (4):201-215, 1996.
Suzuki et al.	2007		Different species: bumble bees	Y. Suzuki, L. G. Kawaguchi, and Y. Toquenaga. Estimating nest locations of bumblebee Bombus ardens from flower quality and distribution. *Ecological Research* 22 (2):220-227, 2007.
Taneyhill	2007		Different species: bumble bees	D. E. Taneyhill. Foraging mechanisms and the currency for models of energy maximization in bumble bees (Hymenoptera : Apidae : Bombus occidentalis). *Entomologia Generalis* 30 (2):119-133, 2007.
Williams and Thomson	1998		Different species: bumble bees	N. M. Williams and J. D. Thomson. Trapline foraging by bumble bees: III. Temporal patterns of visitation and foraging success at single plants. *Behavioral Ecology* 9 (6):612-621, 1998.
Schmid-Hempel et al.	1985	yes	Additional	Schmid-Hempel, P., Kacelnik, A. & Houston, A.I. (1985) Honeybees maximize efficiency by not filling their crop. *Behavioral Ecology and Sociobiology* , 17, 61–66.
Camazine and Sneyd	1991	yes	Additional	Camazine S, Sneyd J (1991) A Model of Collective Nectar Source Selection by Honey Bees: Self-organization Through Simple Rules. *Journal of Theoretical Biology* , 149, 547-571.
de Vries and Biesmeijer	1998, 2002	yes	Additional	De Vries, H. & Biesmeijer, J.C. (1998) Modelling collective foraging by means of individual behaviour rules in honey-bees. Behavioral Ecology and Sociobiology, 44, 109-124.
Sumpter and Pratt	2003	yes	Additional	Sumpter, D.J.T. & Pratt, S.C. (2003) A modelling framework for understanding social insect foraging. *Behavioral Ecolology and Sociobiology* . 53, 131–144.
Schmickl and Crailsheim	2004	yes	Additional	Schmickl, T. & Crailsheim, K. (2004) Costs of environmental fluctuations and benefits of dynamic decentralized foraging decisions in honey bees. *Adaptive Behavior* , 12, 263-277
Dornhaus et al.	2006a	yes	Additional	Dornhaus, A., Klügl, F., Oechslein, C., Puppe, F. & Chittka, L. (2006) Benefits of recruitment in honey bees: effects of ecology and colony size in an individual-based model. *Behavioral Ecology* , 17, 336-344.

Supporting Information: Becher et al: Modelling Honeybee colonies

S1b: Foraging models

Authors	Year	reviewed	Comment	Full reference
Dornhaus et al.	2006b	yes	Additional	Dornhaus, A., Collins, E.J., Dechaume-Moncharmont, F.-X., Houston, A.I., Franks, N.R. & McNamara, J.M. (2006) Paying for information: partial loads in central place foragers. *Behavioral Ecology and Sociobiology* 61,151–161.
Beekman et al.	2007	yes	Additional	Beekman, M., Gilchrist, A.L., Duncan, M., Sumpter, D.J.T. (2007) What makes a honeybee scout? *Behavioral Ecolology and Sociobiology* , 61, 985-995.
Johnson and Nieh	2010	yes	Additional	Johnson, B.R. & Nieh, J.C. (2010) Modeling the adaptive role of negative signaling in honey bee intraspecific competition. *Journal of Insect Behavior* 23, 459-471.
Schmickl et al.	2012	yes	Additional	Schmickl, T., Thenius, R., Crailsheim, K. (2012) Swarm-intelligent foraging in honeybees: benefits and costs of task-partitioning and environmental fluctuations. *Neural Computing and Applications* , 21, 251-268.
