# Supplementary material for: REVIEW: Towards a systems approach for understanding honeybee decline: a stocktaking and synthesis of existing models
Source: J Appl Ecol. 2013 Jun 10;50(4):868–80. doi: 10.1111/1365-2664.12112 (PMC3810709; doi:10.1111/1365-2664.12112)
Supplement: Supplementary file 2 — Table S2. Additional information on the models listed in Table 1: Model output presented (all models), honeybee representation (varroa models), recruitment (foraging models). [file JPE-50-868-s002.pdf]

Table S2. Honeybee models evaluated in this review. Terms used in model output boxes: N = number, i.e. abundance; (t) = through time. **a)** Colony models; **b)** Varroa models; representation of the honeybee is described as Imposed = determined by a fixed pattern; or Emergent = arising during model run. **c)** Foraging models; Recruitment column lists the entities involved in the foraging and recruitment processes: scouts: search new patches, recruits: are recruited to a certain patch by a dancing bee, receivers: accept nectar from successful foragers.

a) Colony models

Reference	Model output presented
Omholt 1986	N brood and worker bees (t), ratio brood/worker bees, peak worker bee abundance, timing of peak abundance of brood and worker bees
deGrandi-Hoffman *et al*. 1989	N adult bees (t), peak adult bee abundance, % worker in colony, colony growth rate, proportion of foragers (t),
Martin 2001[1]	N adult bees (t), mite population (t), age structure of adult bees (t), time to colony death vs. initial number of mites transmitting DWV, size of mite population transmitting APV that is required for colony death (t)
Al Ghamdi & Hoopingarner 2004[2]	N drone and worker brood (t), N adult bees (t), mite population (t)
Thompson *et al*. 2005 & 2007[3]	% reduction in winter size population due to pesticide application at different times of the year, % loss mean colony size
Schmickl & Crailsheim 2007	Life table (number of survivors vs. bee age), N adult bees (t), N brood cells (t), pollen stores (t), honey stores (t), colony weight (t), nectar stores (t), usage honey (t), maximum number/level of: adult bees/ brood cells/ honey stores/ pollens stores, adult bees at end of year, honey stores at end of year, cumulative population (brood and adults) input and losses (t), N larvae cells (t),
Becher *et al*. 2010	N workers (t), initial N of workers needed for survival of the colony, assessment of cost-benefit ratio for improved thermoregulation (‘relative gain’)
Khoury *et al*. 2011	Phase plane in-hive bees/foragers, N bees (t), average age in-hive bees/onset of foraging vs. forager mortality, equilibrium colony size vs. forager mortality, average age of first foraging, average lifespan

[1] Model very similar to DeGrandi-Hoffman et al. (1989).
[2] Honeybee model very similar to DeGrandi-Hoffman et al. (1989), varroa model very similar to Fries et al. (1994).
[3] Model very similar to Wilkinson and Smith (2002).

b) Varroa models

Reference	Model output presented	Honeybee representation
Omholt & Crailsheim 1991	Predicted winter mite populations vs. death rate in summer and the corresponding coefficients of correlation	Imposed: dates for period of worker and drone brood production
Calis, Fries, Ryrie 1999b (an update and extension of Fries et al. 1994)	N mites (t), % mites in drone and worker cells (t), mite mortality (t), effects of reinvasion, acid treatment, drone brood removal, climate on N mites (t)	Imposed: empirical worker and drone brood data (mid-European and neo-tropical conditions), calculation of adults
Fries et al. 1994 (predecessor of Calis et al. 1999)	N mites (t) with/without acaricide treatment, brood removal	Imposed: empirical drone and worker brood data
Boot et al. 1995	Net rate of mite population increase (t), emerging mites from drone cells (t), relative mortality rate while living on adult bees (t); emerging mites from worker cells(t)	Imposed: encounter rates for drone, worker cells
Martin 1998 (predecessor of Martin 2001[4])	N mites (t), mean phoretic period, preference of mites for drone cells (t), mite drop (t), mites (t) under shortened developmental time, acaricide treatment, ratio falling to live mites (t), infestation level (t), % mites in brood cells (t)	Imposed: empirical data on adult workers and drone & worker brood (eggs, larvae, pupae)
Calis, Boot, Beemtsma 1999a	N brood cells needed to trap 95% of mites vs. colony size, mite population (t), predicted mite reduction and effectiveness of treatment	Imposed: colony profile (30,000 worker and brood cells, 4% drone brood)
Wilkinson & Smith 2002	N mites (t), mite population growth rate (t); N mites after one year vs. length of post capping time	Imposed: colony profile for worker and drone brood and adult workers
DeGrandi-Hoffman & Curry 2004 (extension of BEEPOP)	Mite drop (t), N adult bees (t) with/without Varroa and miticide treatment, rate of colony increase (t), % mites killed by miticide (t), mites (t), mites per drone and worker cell (t)	Emergent: based on BEEPOP
Sumpter & Martin 2004	N mites required for epidemic and colony collapse, N healthy and infected bees vs. N mites, N bees after vs. before winter, N bees (t)	Emergent: seasonal egg laying, infection dependent mortality
Vetharaniam & Barlow 2006 (based on Wilkinson & Smith 2002)	N mites (t), effect of increase and decrease of mites' cell invasion rate; N phoretic and virulent mites (t), Nv phoretic and virulent mites vs. % virulent mites, difference in long-term N phoretic and virulent mites vs. % virulent mites, thresholds for % virulent mites to cause damage	Imposed: two colony profiles (short and long season) for worker and drone brood and adult workers
Vetharaniam 2012	Distribution of mites in worker and drones cells, N mites produced vs. N mites invaded, mite population(t)	Imposed: based on bee 'activity level'

[4] Martin 2001 includes a fully-developed varroa model, but is filed under colony models (Tables 1 and 2). Colony dynamics emerges from a fully-developed colony model.

c) Foraging models

Reference	Model output presented	Recruitment
Schmid-Hempel et al. 1985	N visited flowers vs. inter-flower time for empirical data and for modelled maximisation of rate or efficiency; comparison predicted vs. observed N visited flowers for rate and efficiency; sensitivity: N flowers vs. inter-flower time for rate and efficiency, different metabolic costs	No
Camazine & Sneyd 1991	N bees (t) for two feeders with and without switching quality of feeders; N bees (t) at two feeders (foragers, dance followers) for changes in probabilities to dance, to follow a dance, and to abandon a feeder	Recruits
de Vries & Biesmeijer 1998, 2002	N foragers at food source (t) for various combinations of search ability and transmission (dance) accuracy, different abandoning probabilities, two sugar concentrations, and var. workforces	Recruits;<br>2002: also receivers
Dukas & Edelstein-Keshet 1998	% foragers at patch vs. relative distance of patch for three currencies, for solitary & social foragers, for various mortality rates; forager density vs. distance from colony	No
Sumpter & Pratt 2003	Recruitement rate vs. N waiting workers, vs. N dancers; discovery rate vs. N ants on trail; rate to join trail or explore vs. N ants on trail; steady state foraging vs. colony size; rel. productivity vs. investment in workers;	Scouts, recruits,
Higginson & Gilbert 2004	Sensitivity: N flowers vs. % wing damage for search time, mortality and learning rate; N flowers vs. % wing damage for model results and observed data fit; N flowers vs. predation risk; N flowers vs. search time	No
Schmickl & Crailsheim 2004 (HoFoSim)	N foragers (t) at two feeders; net honey gain (t) for different sugar concentrations; net honey gain vs. sugar concentration of alternative feeder; net honey gain (t) for different frequencies of quality fluctuations	Scouts, recruits, receivers
Dornhaus et al. 2006a	Energy gain vs. patch quality vs. N patches (with and without recruitment), recruitment benefit vs. patch quality vs. N patches, energy gain vs. recruitment intensity, sensitivity: recruitment benefits vs. 12 variables	Scouts, recruits
Dornhaus et al. 2006b	Optimal foraging time and effect of reducing load vs. relative reward rate, and vs. duration of superior patch availability	No
Beekman et al. 2007	Proportion of bees (t) (foragers, searchers, dancers, scouts) for high and low profitable food source, probility to find a dance vs. N dancers	Scouts, recruits
Johnson & Nieh 2010	N attacking bees (t) with and without stop signal for different guard recruitment rates, for different levels of rejection of attacked colony.	Scouts, recruits, receivers
Schmickl et al. 2010 (HoFoReSim)	N bees with info (t); foragers (t); % foragers on dominant source vs. rel. N receiver bees; N foragers (t) for different levels of receiver bees and for three feeder scenarios; honey stores (t); costs of environmental fluctuations vs. n receiver bees; honey gain vs. receiver bees.	Scouts, recruits, receivers
