# Supplementary material for: REVIEW: Towards a systems approach for understanding honeybee decline: a stocktaking and synthesis of existing models
Source: J Appl Ecol. 2013 Jun 10;50(4):868–80. doi: 10.1111/1365-2664.12112 (PMC3810709; doi:10.1111/1365-2664.12112)
Supplement: Supplementary file 3 — Table S3. Listing entities and state variables of the reviewed colony models. [file JPE-50-868-s003.pdf]

Table S3. Entities and state variables of colony models. 'Age' refers to age cohorts, characterized by their age in days and number of individuals; N: number, i.e. abundance; Sex: distinction between worker and drone larvae.

<table>
<tr><th>ENTITY / MODEL</th><th>Omholt 1986</th><th>deGrandi-Hoffman et al. 1989</th><th>Martin 2001</th><th>AlGhamdi & Hoopingarner 2004</th><th>Thompson et al. 2005/2007</th><th colspan="2">Schmickl & Crailsheim 2007</th><th>Becher et al. 2010</th><th>Khoury et al. 2011</th></tr>
<tr><td>Winter bees</td><td>N</td><td></td><td></td><td></td><td></td><td colspan="2"></td><td>Age</td><td></td></tr>
<tr><td>Queen</td><td></td><td>Age, sperm</td><td></td><td>Age</td><td></td><td colspan="2"></td><td></td><td></td></tr>
<tr><td>Eggs</td><td>N[1]</td><td>Age</td><td>Age</td><td>Age, sex</td><td></td><td colspan="2">Age</td><td rowspan="3">Age</td><td></td></tr>
<tr><td>Larvae (brood)</td><td>N</td><td rowspan="2">Age, sex</td><td>Age, sex</td><td rowspan="2">Age, sex</td><td rowspan="2">N, sex</td><td colspan="2">Age</td><td></td></tr>
<tr><td>Pupae (sealed brood)</td><td>N</td><td>Age, sex</td><td colspan="2">Age</td><td></td></tr>
<tr><td>Drones</td><td rowspan="3">N</td><td>Age</td><td>Age</td><td>Age</td><td></td><td colspan="2"></td><td rowspan="2">Age
Temperature at emergence</td><td rowspan="2">N</td></tr>
<tr><td>In-hive bees</td><td>Age</td><td rowspan="2">Age</td><td rowspan="2">Age</td><td>N[4]</td><td rowspan="2">N</td><td>N nurses
N nectar processors</td></tr>
<tr><td>Foragers</td><td>Age</td><td>N</td><td>N pollen foragers[2]
N nectar foragers</td><td>Age</td><td>N</td></tr>
<tr><td>Comb</td><td></td><td></td><td></td><td></td><td></td><td colspan="2">N of empty and brood cells
Stores of nectar, pollen, honey</td><td>N of empty and brood cells</td><td></td></tr>
<tr><td>Environment</td><td></td><td>Temperature
Photoperiod
Windspeed
Rainfall</td><td>Temperature
Photoperiod
Windspeed
Rainfall</td><td>Temperature
Photoperiod</td><td></td><td colspan="2">Season
Rain
Temperature</td><td></td><td></td></tr>
<tr><td>Invaded mites</td><td rowspan="2"></td><td rowspan="2"></td><td>N</td><td></td><td></td><td colspan="2" rowspan="2"></td><td rowspan="2"></td><td rowspan="2"></td></tr>
<tr><td>Phoretic mites</td><td>N</td><td>N</td><td></td></tr>
</table>

[1] No equations provided
[2] Distinguished between workforce (available) and active (demand driven)
