# Supplementary material for: REVIEW: Towards a systems approach for understanding honeybee decline: a stocktaking and synthesis of existing models
Source: J Appl Ecol. 2013 Jun 10;50(4):868–80. doi: 10.1111/1365-2664.12112 (PMC3810709; doi:10.1111/1365-2664.12112)
Supplement: Supplementary file 4 — Table S4. Listing processes and feedbacks of the reviewed colony models. [file JPE-50-868-s004.pdf]

Table S4. Processes and feedbacks in the colony models. For each colony model, all main processes are listed, including the factors on which the process depends. These factors can be complex in themselves. If a process includes no factor, it is represented via a constant parameter. Feedbacks between processes and state variables of the model are underlined.

**Omholt 1986**	**deGrandi-Hoffman *et al.* 1989**	**Martin 2001**	**AlGhamdi & Hoopingarner 2004**	**Thompson *et al.* 2005/2007**	**Schmickl & Crailsheim 2007**	**Becher *et al.* 2010**	**Khoury *et al.* 2011**
Egg laying<br>- Queen state<br>- N bees<br><br>Brood mortality<br><br>Development<br><br>In-hive bee mortality<br>- Season	Queen ageing<br><br>Egg laying<br>- Queen age<br>- Weather<br>- N foragers<br><br>Fertilization<br>- N spermatozoa<br>- Photoperiod<br>- N foragers<br><br>Brood mortality<br><br>Development<br><br>In-hive bee mortality<br><br>Foraging<br>- Weather<br><br>Forager mortality	Egg laying<br>- Weather<br>- N adult bees<br><br>Brood mortality<br>- N adult bees<br>- Varroa<br><br>Development<br><br>In-hive bee mortality<br>- Age<br>- Trend in egg production<br>- Virus<br><br>Mite dynamics	Queen ageing<br><br>Egg laying<br>- Queen age<br>- Weather<br>- N adult bees<br><br>Brood mortality<br><br>Development<br><br>In-hive bee mortality<br>- Season<br>- Mites<br><br>Mite dynamics	Egg laying<br>- Seasonal factor<br>- Pesticide exposure<br><br>Brood mortality<br>- N in-hive bees<br><br>Development<br><br>Adult bee mortality<br>- Seasonal factor<br>- Pesticides<br><br>Foraging<br>- Premature foraging due to pesticides	Egg laying<br>- Seasonal factor<br>- N empty cells<br><br>Brood mortality<br>- Eggs, pupae: const<br>- Larvae: Pollen store, N larvae, N adults<br><br>Larvae cannibalism<br>- Pollen store<br>- N larvae, adults<br><br>Development<br><br>In-hive bee mortality<br>- Task-specific<br>- Colony structure<br>- Stores nectar, pollen<br><br>Foraging<br>- Weather<br>- Demand pollen. nectar<br><br>Nursing<br>- N brood<br>- Age larvae<br><br>Food processing<br>- Stored nectar<br><br>Use of pollen, nectar,	Egg laying<br>- N empty and bood cells<br>- Temperature in brood cells<br><br>Development<br><br>Heating of brood cells<br>- N in-hive bees<br><br>In-hive bee mortality<br><br>Foraging<br>- Age at first foraging determined by temperature during development<br><br>Forager mortality	Eclosion<br>- N in-hive and foragers<br><br>Foraging<br>- Recruitment to foraging depends on fraction of foragers<br><br>Forager mortality

					honey<br>- Stores<br>- N larvae, adults<br>Forager mortality<br><br>Forage availability<br>- Seasonal factor		
