# Supplementary material for: REVIEW: Towards a systems approach for understanding honeybee decline: a stocktaking and synthesis of existing models
Source: J Appl Ecol. 2013 Jun 10;50(4):868–80. doi: 10.1111/1365-2664.12112 (PMC3810709; doi:10.1111/1365-2664.12112)
Supplement: Supplementary file 5 — Appendix S5. Discussion of the complexity, and importance of feedback loops of the reviewed models [file JPE-50-868-s005.docx]

**Appendix S5:** discussion of the complexity and importance of feedback loops of the reviewed models

How does the complexity and realism of reviewed models help us understand colony losses?

**Complexity:** Traditional modelling philosophy in ecology emphasizes the costs of increased model complexity in terms of parameterization, understanding, testing, and generality (e.g., May 1974). However, honeybee colony dynamics and foraging are controlled by a complex set of feedback mechanisms. Simple models cannot assess how such feedback mechanisms interact. For instance, the simple differential equation model of Khoury, Myerscough & Barron (2011) focuses on one important feedback mechanism (Table S4), but whether or not it is this very mechanism that explains observed phenomena remains an open question because other mechanisms might lead to similar model behaviour. Increased model complexity and richness in model structure and mechanisms enables the comparison of the model to more than just one aggregated level of observations and allows independent predictions of new patterns that were not considered during model design and parameterization (DeAngelis & Mooij 2003, Grimm *et al.* 2005, Grimm & Railsback 2012). For instance, Martin’s (2001) model predicts colony dynamics, the patterns of occurrence of APV and DWV in varroa-infested, and the number of mites infected with APV needed to kill a colony. Other examples are Schmickl & Crailsheim (2007) (dynamics of nectar stores and honey usage; oscillations in number of unsealed brood cells for hive with smaller capacities), Becher *et al.* 2010 (existence of a threshold initial population size for colony survival) and Omholt 1986 (ratio brood/adult bees vs. initial colony size).

**Feedback mechanisms:** Ratnieks & Carreck (2010) have suggested that the recent observations of honeybee mortalities might be due to the impact of stressors reaching some catastrophic threshold where the colony’s resilience, or buffer mechanisms, have reached the limits of their capacity. Understanding the feedback mechanisms and resulting resilience and its capacity is therefore key to understanding and managing honeybee colony numbers. Most of the models include only one or a few feedback loops because the purpose of the models was to explore those mechanisms. In contrast, HoPoMo (Schmickl & Crailsheim 2007) includes many more feedbacks than any other model (see their Fig. 12) but so far has mainly been analysed under conditions where resilience works. It would be interesting, and is mandatory for any model addressing colony collapse, to also perform “resilience analyses” where the system is stressed or disturbed in various ways. This would provide insights about the relative significance of different feedbacks, their interactions, and their limits.

**Additional references:**

DeAngelis, D.L. & Mooij, W.M. (2005) Individual-based modeling of ecological and evolutionary processes*. Annual Review of Ecology and Systematics*, **36**, 147–168

Grimm, V., Revilla, E., Berger, U., Jeltsch, F., Mooij, W.M., Railsback, S.F., Thulke, H.-H., Weiner, J., Wiegand, T. & DeAngelis, D.L. (2005) Pattern-oriented modeling of agent-based complex systems: lessons from ecology. *Science*, **310**, 987–991.

May, R.M. (1974) *Stability and Complexity in Model Ecosystems*, 2nd edn. Princeton University Press, Princeton, NJ.
